# Sinkhorn Distributionally Robust Conditional Quantile Prediction with Fixed Design

**DOI:** 10.3390/e27060557

**Published:** 2025-05-25

**Authors:** Guohui Jiang, Tiantian Mao

**Affiliations:** Department of Statistics and Finance, School of Management, University of Science and Technology of China, Hefei 230052, China; ghjiang@mail.ustc.edu.cn

**Keywords:** distributionally robust optimization, conditional quantile prediction, Sinkhorn distance

## Abstract

This paper proposes a novel data-driven distributionally robust framework for conditional quantile prediction under the fixed design setting of the covariates, which we refer to as Sinkhorn distributionally robust conditional quantile prediction. We derive a convex programming dual reformulation of the proposed problem and further develop a conic optimization reformulation for the case with finite support. Our method’s superior performance is demonstrated through several numerical experiments, highlighting its effectiveness in practical applications.

## 1. Introduction

Quantile regression [1] aims to estimate the value of a specific quantile of a given random variable. During the past few decades, quantile regression has been widely applied in various fields of academic research, including economics [2], clinical medicine [3], and environmental science [4], among others. In the financial field, for example, the 1% or 5% quantile describing the left tail of the distribution of the profit and loss account (the so-called Value-at-Risk (VaR)) is of interest to risk managers [5].

Under the random design framework [1,6], theoretical performance bounds are established by assuming that the data samples are independent and identically distributed (i.i.d.). Although this assumption holds in some cases, it is often unrealistic in practice, as external features may be predetermined or designed, preventing data samples from being treated as realizations of a random variable from an underlying distribution. This phenomenon of predetermined or designed external features is known as fixed design in regression analysis [7].

Common methods for solving conditional quantile prediction problems in the random design include sample average approximation (SAA) [8]. SAA has the advantage of strong applicability, but it is limited by increasing computational and storage costs as the sample size grows. However, when using the fixed design setting, the solution obtained using the SAA method may not be robust [9]. To achieve a robust yet not overly conservative solution, one can consider the distributionally robust optimization (DRO) approach [10,11]. DRO, which serves as a modeling paradigm for decision-making under model uncertainty, seeks to identify an optimal decision by minimizing the worst-case (or adversarial) outcome over a set of plausible probability distributions, commonly referred to as the ambiguity set.

The construction of the ambiguity set plays a crucial role in the performance of the DRO method. In the literature, there are two main approaches to constructing ambiguity sets. The first approach involves defining the ambiguity sets based on descriptive statistics, such as moment conditions [11,12,13], shape constraints [14,15], marginal distributions [16,17], etc. The second approach, which has become popular in recent years, considers distributions within a pre-specified statistical distance from a nominal distribution. Usually, the nominal distribution is the empirical distribution of the data samples. Commonly used statistical distances in the literature include ϕ-divergence [18,19,20], Wasserstein distance [21,22,23], maximum mean discrepancy [24], and Sinkhorn distance [25,26,27,28].

Among all metric-based ambiguity sets, the Kullback–Leibler (KL) ambiguity set received relatively early attention. However, as argued in [21], when the ambiguity set is centered at the discrete empirical distribution and the unknown true distribution is absolutely continuous with respect to the Lebesgue measure, any KL ambiguity set will necessarily exclude the true distribution. This occurs because the unknown true distribution is a continuous distribution that cannot assign positive probability mass to each training sample. In contrast, the Wasserstein ambiguity set naturally contains both discrete and continuous distributions, thereby overcoming the limitation of the KL ambiguity set. Subsequently, although Wasserstein distance is relatively popular in DRO research, it still has certain limitations. Wasserstein DRO is typically tractable only under stringent conditions on the loss function (see Table 1 in [27]), and for data-driven Wasserstein DRO, where the nominal distribution is finitely supported (usually the empirical distribution), the worst-case distribution is discrete and supported on, at most, N+1 points, although the underlying true distribution in many practical applications may be continuous [23]. This raises concerns about whether Wasserstein DRO hedges the correct family of distributions and whether it may lead to overly conservative performance.

To address these potential issues while maintaining the advantages of Wasserstein DRO, this paper adopts the Sinkhorn distance [25,26] to construct the ambiguity set. Sinkhorn distance, a smoothed variant of the Wasserstein distance, is defined as the minimal transport cost between two distributions in an entropy-regularized optimal transport problem. This design improves the computational complexity of Wasserstein distance, with demonstrated efficiency gains enabling widespread applications in domain adaptation [29,30], generative modeling [31,32], and dimensionality reduction [33,34,35].

Sinkhorn DRO refers to constructing an ambiguity set of distributions using Sinkhorn distance, finding the worst-case distribution that maximizes the loss within this ambiguity set, and subsequently finding the optimal decision within the decision set that minimizes the loss corresponding to the worst-case distribution. As argued by [27], computing the Sinkhorn distance and solving the Sinkhorn DRO problem are two different computational tasks. From the perspective of dual formulations, the former is a standard stochastic optimization problem, whereas the latter is a conditional stochastic optimization [36]. In the latter case, computing unbiased gradients becomes extremely challenging. Thus, the Sinkhorn DRO problem is computationally non-trivial.

To the best of our knowledge, research on Sinkhorn DRO is still in its early stages, with representative theoretical works including [27] and [28]. Compared to the Wasserstein DRO, its advantages mainly lie in the fact that the dual problem of Sinkhorn DRO is computationally tractable for a broader class of loss functions, cost functions, nominal distributions, and probability supports [27], and its worst-case distribution is absolutely continuous with respect to a pre-specified reference measure, such as the Lebesgue or counting measure. This characteristic of Sinkhorn DRO highlights its flexibility as a modeling choice and provides a more realistic representation of uncertainty that better aligns with the underlying true distribution in practical scenarios [27].

Currently, under the fixed design setting, the authors of [9] proposed a distributionally robust conditional quantile prediction problem based on a type-1 Wasserstein ambiguity set, establishing the finite sample guarantee and asymptotic consistency. Building on this work, Ref. [37] employed the type-*p* Wasserstein ambiguity set, thereby generalizing the original problem. Recognizing the advantages of Sinkhorn DRO over Wasserstein DRO, in this paper, we propose a Sinkhorn distributionally robust conditional quantile prediction (DRCQP) problem under the fixed design setting and establish its strong dual reformulation to ensure computational tractability. When the support is finite, the dual form reduces to an exponential cone program. We further investigate the theoretical relationships between our proposed problem and some classic DRCQP problems. Finally, through empirical analysis, we evaluate the out-of-sample performance of our proposed method and conduct sensitivity analyses of the key parameter η, thereby providing practical references for real-world applications.

The remainder of the paper is structured as follows. Section 2 presents the preliminary knowledge, including the standard linear regression model and the conditional quantile regression model. Section 3 introduces the definition of Sinkhorn distance and establishes the Sinkhorn DRCQP problem under the fixed design setting. Section 4 aims to tackle the proposed problem by providing an equivalent tractable reformulation under some mild assumptions, along with a conic optimization reformulation developed for finite support cases. Section 5 evaluates the performance of our DRO method through numerical experiments. Finally, Section 6 concludes the paper with a summary of key findings.

**Notations**: Let $R^{n}$ denote the *n*-dimensional Euclidean space. For a positive integer *N*, let [N] represent the set {1,2,…,N}. For a measurable set Z, denote the set of measures (not necessarily probability measures) as M(Z) on Z, and let P(Z) denote the set of probability measures on Z. Given a probability distribution P and a measure μ, we denote P≪μ if P is absolutely continuous with respect to μ. P⊗Q denotes the product measure of two probability distributions P and Q. Given a measure μ∈M(Z) and a measurable variable f:Z→R, we write $E_{z∼\mu }\left[f\left(z\right)\right]$ for $\int_{R}f\left(z\right)d\mu \left(z\right)$. For a given element *x*, denote the one-point probability distribution as $\delta_{z}$ supported on {z}.

## 2. Preliminaries

In this paper, we consider Y∈R as the response variable of interest and $\left(X_{1},\ldots ,X_{p}\right)\in R^{p}$ as the vector of explanatory variables. The standard linear regression model without an intercept term is

(1) $$Y=\beta_{1}X_{1}+\beta_{2}X_{2}+\cdots +\beta_{p}X_{p}+\varepsilon ,$$

where $\beta ={\left(\beta_{1},\ldots ,\beta_{p}\right)}^{⊤}\in R^{p}$ is the vector of unknown regression parameters, and ε∈R is a random variable of residuals. We assume that ε is independent of $\left(X_{1},\cdots ,X_{p}\right)$ with zero mean and unknown variance $\sigma^{2}$.

The ordinary least squares (OLSs) method is the classic approach to estimating the unknown β, but it is highly sensitive to outliers and performs poorly when ε follows the non-Gaussian distribution [1]. To overcome these limitations, Koenker and Basset [1] proposed quantile regression, which minimizes the asymmetric absolute deviations to characterize how explanatory variables affect different quantiles of the conditional distribution of the response variable *Y*. This research originated from the definition of the α-quantile $Q_{\alpha }\left(Y\right)$ of the response variable *Y*:

(2) $$Q_{\alpha }\left(Y\right)=\inf\left\{q\in R:P\left(Y\leq q\right)\geq \alpha \right\},0<\alpha <1.$$

In addition to (2), $Q_{\alpha }\left(Y\right)$ can also be expressed as the solution to the following stochastic optimization problem:

(3) $$Q_{\alpha }\left(Y\right)=\underset{q\in R}{\arg\min}E\left[\rho_{\alpha }\left(Y-q\right)\right],$$

where $\rho_{\alpha }$ is the quantile loss function (also called the check function), defined as

$$\rho_{\alpha }\left(u\right)=u(\alpha -1_{\left(u\leq 0\right)})=\left\{\begin{array}{cc} \alpha u, & \;\mathrm{if}\;u>0,\\ (\alpha -1)u, & \;\mathrm{if}\;u\leq 0. \end{array}\right.$$

which assigns different weights to positive and negative residuals.

In the fixed design setting, we focus on the out-of-sample performance with certain feature vectors $\left(X_{1},\cdots ,X_{p}\right)={\left(x_{1},\cdots ,x_{p}\right)}^{f}:=x^{f}$ (Here, the superscript “*f*” in $x^{f}$ represents the fixed design.). According to the linear problem (1) and the independence between $\left(X_{1},\cdots ,X_{p}\right)$ and ε, the α-th conditional quantile $Q_{\alpha }\left(Y|x^{f}\right)$ of the response variable *Y* takes the following form:

$$Q_{\alpha }\left(Y|x^{f}\right)=Q_{\alpha }\left(\beta^{⊤}x^{f}+\varepsilon |x^{f}\right)=\beta^{⊤}x^{f}+Q_{\alpha }\left(\varepsilon \right)=\beta^{⊤}x^{f}+s_{\alpha },$$

where $s_{\alpha }:=Q_{\alpha }\left(\varepsilon \right)$ is defined as the α-th quantile of ε. Consider *N* observed samples ${\left\{\left(x_{i}^{f},y_{i}\right)\right\}}_{i=1}^{N}$ generated from model (1). According to [1], for α∈(0,1), the α-th regression quantile is defined as the solution to the following problem:

$$\min_{\tilde{\beta }\in R^{p},\;s\in R}\sum_{i=1}^{N}\rho_{\alpha }\left(y_{i}-{\tilde{\beta }}^{⊤}x_{i}^{f}-s\right).$$

The aforementioned quantile regression problem can be viewed as applying the sample average approximation (SAA) method to solve the conditional stochastic optimization problem in (3). Under the random design setting, the SAA method performs well since the empirical distribution converges to the true distribution as the sample size *N* increases. However, under the fixed design setting, this convergence cannot be guaranteed, resulting in the deterioration of estimation efficiency. This motivates us to find more effective methods to solve the conditional quantile regression problem under the fixed design setting.

## 3. Problem Setup

### 3.1. Sinkhorn Distance

The core of distributionally robust optimization (DRO) lies in selecting a suitable ambiguity set. Such a set must balance computational tractability with practical interpretability. It should be sufficiently rich to cover relevant distributions yet exclude unnecessary ones that may lead to overly conservative decisions. Recently, the Sinkhorn distance has gained significant attention for constructing ambiguity sets. Its definition is as follows:

**Definition** **1** (Sinkhorn distance, [25,26,27])**.** *Let Z be a measurable set. Consider distributions P,Q∈P(Z), and let μ,ν∈M(Z) be two reference measures such that P≪μ,Q≪ν. For the entropic regularization parameter η≥0, the Sinkhorn distance between two distributions P and Q is defined as*

$$W_{\eta }\left(P,Q\right)=\underset{\gamma \in \Gamma (P,Q)}{\inf}\left\{E_{(z,z^{\prime })∼\gamma }\left[d{\left(z,z^{\prime }\right)}^{p}\right]+\eta H\left(\gamma |\mu ⊗\nu \right)\right\},$$

*where Γ(P,Q) is defined as the set of all Borel probability distributions on Z×Z with marginal distributions P and Q, $d{\left(z,z^{\prime }\right)}^{p}$ denoting the cost function, and H(γ|μ⊗ν) denotes the relative entropy (also called Kullback–Leibler divergence, KL divergence) of γ with respect to the product measure μ⊗ν:*

$$H\left(\gamma |\mu ⊗\nu \right)=E_{(z,z^{\prime })∼\gamma }\left[\log\left(\frac{d\gamma (z,z^{\prime })}{d\mu \left(z\right)d\nu \left(z^{\prime }\right)}\right)\right].$$

When η=0, the Sinkhorn distance $W_{\eta }\left(P,Q\right)$ reduces to the classic Wasserstein metric, and as η goes to infinity, the entropic regularization term dominates; the distance increasingly emphasizes the entropy of the coupling rather than the transport cost. In this paper, we study a distributionally robust conditional quantile prediction problem, which is formulated as a one-dimensional minmax optimization problem. This leads us to focus on Z⊆R, and we take the standard distance $d\left(z,z^{\prime }\right)=\left|z-z^{\prime }\right|$.

Based on the definition of the Sinkhorn distance, we introduce the Sinkhorn ball centered on a reference distribution to effectively capture and characterize the distributional ambiguity in our analysis.

**Definition** **2.** *For a fixed distribution P∈P(Z) and r>0, the Sinkhorn ball $B_{r,\eta }\left(P\right)$ of radius r centered on P is defined as*

(4) $$B_{r,\eta }\left(P\right):=\left\{Q\in P\left(Z\right)\;|\;W_{\eta }\left(P,Q\right)\leq r\right\}.$$

Due to the convex entropic regularizer in $W_{\eta }\left(P,Q\right)$ with respect to P[38], the Sinkhorn distance $W_{\eta }\left(P,Q\right)$ is convex in P, i.e., $W_{\eta }\left(P,\lambda Q_{1}+\left(1-\lambda \right)Q_{2}\right)\leq \lambda W_{\eta }\left(P,Q_{1}\right)+\left(1-\lambda \right)W_{\eta }\left(P,Q_{2}\right)$ for all probability distributions $Q_{1},Q_{2}\in P\left(Z\right)$ and all 0≤λ≤1. Therefore, the Sinkhorn ball $B_{r,\eta }\left(P\right)$ is a convex set.

### 3.2. Sinkhorn Distributionally Robust Conditional Quantile Prediction with Fixed Design

Under the fixed design setting, the optimal solution obtained by the SAA method for conditional quantile prediction is not robust [9]. To obtain a robust yet not overly conservative solution, we employ the DRO approach for modeling conditional quantile prediction, building upon similar methodologies proposed in [9]. The corresponding mathematical formulation is presented as follows:

(5) $$\min_{\tilde{\beta }\in R^{p},\;s\in R}\;\underset{P_{Y}\in P_{Y}}{\sup}E_{P_{Y}}\left[\rho_{\alpha }\left(Y-{\tilde{\beta }}^{⊤}x^{f}-s\right)\right],$$

where $P_{Y}$ is a distribution of *Y* conditional on $x^{f}$, and $P_{Y}$ is an ambiguity set constructed from the observed data points ${\left\{\left(x_{i}^{f},y_{i}\right)\right\}}_{i=1}^{N}$ that are generated by model (1).

To transfer the distributional uncertainty from the response variable *Y* to the residual term $\tilde{\varepsilon }$ and thereby simplify problem (5), we define $\tilde{\varepsilon }=Y-{\tilde{\beta }}^{⊤}x^{f}=\varepsilon +{\left(\beta -\tilde{\beta }\right)}^{⊤}x^{f}$. Thus, problem (5) can be rewritten as

$$\min_{\tilde{\beta }\in R^{p},\;s\in R}\;\underset{P_{\tilde{\varepsilon }}\in P_{\tilde{\varepsilon }}}{\sup}E_{P_{\tilde{\varepsilon }}}\left[\rho_{\alpha }\left(\tilde{\varepsilon }-s\right)\right],$$

where $P_{\tilde{\varepsilon }}$ and $P_{\tilde{\varepsilon }}$ are, respectively, the candidate distribution and ambiguity set of $\tilde{\varepsilon }$.

Note that $P_{\tilde{\varepsilon }}$ depends on the unknown $\tilde{\beta }$. Following [9], we first estimate $\tilde{\beta }$ using the OLS estimator ${\hat{\beta }}_{N}^{\mathrm{OLS}}$, which can be expressed as

$${\hat{\beta }}_{N}^{\mathrm{OLS}}={\left({\left(X^{f}\right)}^{⊤}X^{f}\right)}^{-1}{\left(X^{f}\right)}^{⊤}y,$$

where *N* samples ${\left\{\left(x_{i},y_{i}\right)\right\}}_{i=1}^{N}$ are observed from model (1), and $X^{f}$ is the fixed design matrix with a sequence of (row) vectors $\left\{{\left(x_{1}^{f}\right)}^{⊤},\cdots ,{\left(x_{N}^{f}\right)}^{⊤}\right\}$ and $y={\left(y_{1},\cdots ,y_{N}\right)}^{⊤}$. Notably, although the row vectors $\left\{{\left(x_{1}^{f}\right)}^{⊤},\cdots ,{\left(x_{N}^{f}\right)}^{⊤}\right\}$ are not i.i.d., we still assume the fixed design matrix $X^{f}$ satisfies Assumptions 1 and 2 in [9]; thus, ${\hat{\beta }}_{N}^{\mathrm{OLS}}$ is a consistent estimator of $\tilde{\beta }$.

We define $\varepsilon^{\prime }=\varepsilon +{\left(\beta -{\hat{\beta }}_{N}^{\mathrm{OLS}}\right)}^{⊤}x^{f}$ with an unknown distribution $P_{\varepsilon^{\prime }}$, which has the corresponding samples ${\left\{\varepsilon_{i}^{\mathrm{OLS}}\right\}}_{i=1}^{N}$ with $\varepsilon_{i}^{\mathrm{OLS}}=y_{i}-{\left({\hat{\beta }}_{N}^{\mathrm{OLS}}\right)}^{⊤}x_{i}^{f}$. Its empirical distribution ${\hat{P}}_{N}\left(\varepsilon^{\mathrm{OLS}}\right)=\frac{1}{N}\sum_{i=1}^{N}\delta_{\varepsilon_{i}^{\mathrm{OLS}}}$ leads to the Sinkhorn ball $B_{r,\eta }\left({\hat{P}}_{N}\left(\varepsilon^{\mathrm{OLS}}\right)\right)$, where $B_{r,\eta }\left(P\right)$ is defined by (4). With this clarification, we now formally introduce our distributionally robust conditional quantile prediction problem based on the Sinkhorn ball:

(6) $$\min_{s\in R}\;\underset{P_{\varepsilon^{\prime }}\in B_{r,\eta }\left({\hat{P}}_{N}\left(\varepsilon^{\mathrm{OLS}}\right)\right)}{\sup}E_{P_{\varepsilon^{\prime }}}\left[\rho_{\alpha }\left(\varepsilon^{\prime }-s\right)\right],$$

where $P_{\varepsilon^{\prime }}$ and $B_{r,\eta }\left({\hat{P}}_{N}\left(\varepsilon^{\mathrm{OLS}}\right)\right)$ denote the candidate distribution and the Sinkhorn uncertainty set of $\varepsilon^{\prime }$, respectively. We refer to problem (6) as a Sinkhorn distributionally robust conditional quantile prediction (DRCQP) problem. In the remainder of this paper, we will continue to study this problem and present its tractable reformulation.

## 4. Tractable Reformulation

In this section, we focus on the Sinkhorn DRCQP problem (6), which is intractable due to the inner maximization problem involving uncountable candidate distributions within the Sinkhorn ambiguity set. Thus, we provide a strong dual reformulation that effectively transforms this problem into a finite-dimensional optimization problem. Moreover, under the assumption of a finite support, problem (6) can be reformulated as an equivalent conic optimization problem, which facilitates its analysis and solution. Finally, we provide the theoretical connections between problem (6) and other formulations studied in the machine learning literature.

Following the discussion in Remark 2 of [27], we take the reference measure $\mu ={\hat{P}}_{N}\left(\varepsilon^{\mathrm{OLS}}\right)$ and let ν be the Lebesgue measure. Other options for reference measures are discussed in Remark 2 of [27]. For notational clarity, we define

(7) $$\bar{r}:=r+\frac{\eta }{N}\sum_{i=1}^{N}\log\left(\int_{R}e^{-|\varepsilon_{i}^{\mathrm{OLS}}-\varepsilon^{\prime }{|}^{p}/\eta }d\varepsilon^{\prime }\right).$$

To reformulate problem (6), we make the following assumption:

**Assumption** **1.** *(i)* *$\nu \left\{z:0\leq |\varepsilon^{\mathrm{OLS}}-\varepsilon^{\prime }{|}^{p}<\infty \right\}=1$ for ${\hat{P}}_{N}$-almost every $\varepsilon^{\mathrm{OLS}};$*

*(ii)*  *$\int_{R}e^{-|\varepsilon^{\mathrm{OLS}}-\varepsilon^{\prime }{|}^{p}/\eta }d\varepsilon^{\prime }<\infty$ for ${\hat{P}}_{N}$ almost every $\varepsilon^{\mathrm{OLS}};$*
*(iii)*  *Z is a measurable space, and the objective function in problem* (6) *is measurable;*
*(iv)*  *For every joint distribution γ on Z×Z with a first marginal distribution ${\hat{P}}_{N}\left(\varepsilon^{\mathrm{OLS}}\right)$, it has a regular conditional distribution $\gamma_{\varepsilon^{\mathrm{OLS}}}$, given that the value of the first marginal equals $\varepsilon^{\mathrm{OLS}}.$*

Assumption 1 is commonly imposed to ensure both the Sinkhorn distance $W_{\eta }\left(P,Q\right)$ and the expected loss $E_{P_{\varepsilon^{\prime }}}\left[\rho_{\alpha }\left(\epsilon^{\prime }-s\right)\right]$ are well-defined. For a more detailed discussion of this assumption, we refer to [27]. Given Assumption 1, we can derive a convex dual reformulation of problem (6).

**Theorem** **1.** *Let the reference measure $\mu ={\hat{P}}_{N}\left(\varepsilon^{\mathrm{OLS}}\right)$, ν be the Lebesgue measure in R and $\bar{r}\geq 0$. Under Assumption 1, problem* (6) *admits the following equivalent reformulation:*

(8) $$\min_{s\in R,\;\lambda \geq 0}\left\{F\left(s,\lambda \right)≜\lambda \bar{r}+\frac{\lambda \eta }{N}\sum_{i=1}^{N}\log\left(E_{Q_{i}\left(\varepsilon^{\prime }\right)}\left[e^{\rho_{\alpha }\left(\varepsilon^{\prime }-s\right)/\left(\lambda \eta \right)}\right]\right)\right\},$$

*where $\bar{r}$ is given by* (7)*, and the kernel probability distribution $Q_{i}\left(\varepsilon^{\prime }\right)$ is given by*

(9) $$\frac{dQ_{i}\left(\varepsilon^{\prime }\right)}{d\varepsilon^{\prime }}=\frac{e^{-|\varepsilon_{i}^{\mathrm{OLS}}-\varepsilon^{\prime }{|}^{p}/\eta }}{\int_{R}e^{-|\varepsilon_{i}^{\mathrm{OLS}}{-\zeta |}^{p}/\eta }d\zeta },\;\;\;i\in \left[N\right].$$

**Proof of Theorem** **1.** We begin by addressing the inner maximization problem in (6),

(10) $$\underset{P_{\varepsilon^{\prime }}\in B_{r,\eta }\left({\hat{P}}_{N}\left(\varepsilon^{\mathrm{OLS}}\right)\right)}{\sup}E_{P_{\varepsilon^{\prime }}}\left[\rho_{\alpha }\left(\varepsilon^{\prime }-s\right)\right].$$

For a fixed s∈R, problem (10) is equivalent to

(11) $$\begin{array}{cc} \underset{P_{\varepsilon^{\prime }}\in P\left(R\right)}{\sup}\; & E_{P_{\varepsilon^{\prime }}}\left[\rho_{\alpha }\left(\varepsilon^{\prime }-s\right)\right]\\ s.t.\; & W_{\eta }\left({\hat{P}}_{N}\left(\varepsilon^{\mathrm{OLS}}\right),P_{\varepsilon^{\prime }}\right)\leq r. \end{array}$$

According to the definition of the Sinkhorn distance $W_{\eta }\left({\hat{P}}_{N}\left(\varepsilon^{\mathrm{OLS}}\right),P_{\varepsilon^{\prime }}\right)$ with ν taken as the Lebesgue measure on R (i.e., $d\nu \left(\varepsilon^{\prime }\right)=d\varepsilon^{\prime }$), problem (11) admits the following equivalent formulation:

(12) $$\begin{array}{cc} \underset{\gamma \in P(R\times R)}{\sup}\; & E_{P_{\varepsilon^{\prime }}}\left[\rho_{\alpha }\left(\varepsilon^{\prime }-s\right)\right]\\ s.t.\; & E_{(\varepsilon^{\mathrm{OLS}},\varepsilon^{\prime })∼\gamma }\left[|\varepsilon^{\mathrm{OLS}}-\varepsilon^{\prime }{|}^{p}+\eta \log\left(\frac{d\gamma (\varepsilon^{\mathrm{OLS}},\varepsilon^{\prime })}{d{\hat{P}}_{N}\left(\varepsilon^{\mathrm{OLS}}\right)\;d\varepsilon^{\prime }}\right)\right]\leq r, \end{array}$$

where $\varepsilon^{\mathrm{OLS}}∼{\hat{P}}_{N}\left(\varepsilon^{\mathrm{OLS}}\right)$ and $\varepsilon^{\prime }∼P_{\varepsilon^{\prime }}$. The joint distribution $\gamma (\varepsilon^{\mathrm{OLS}},\varepsilon^{\prime })$ has marginal distributions ${\hat{P}}_{N}\left(\varepsilon^{\mathrm{OLS}}\right)$ and $P_{\varepsilon^{\prime }}$, respectively. According to the disintegration theorem in [39], we represent the joint distribution γ such that $d\gamma \left(\varepsilon^{\mathrm{OLS}},\varepsilon^{\prime }\right)=d{\hat{P}}_{N}\left(\varepsilon^{\mathrm{OLS}}\right)\;d\gamma_{\varepsilon^{\mathrm{OLS}}}\left(\varepsilon^{\prime }\right)$ holds for any $\left(\varepsilon^{\mathrm{OLS}},\varepsilon^{\prime }\right)$, where $\gamma_{\varepsilon^{\mathrm{OLS}}}$ is the conditional distribution of $\varepsilon^{\prime }$, given the first marginal of γ equals $\varepsilon^{\mathrm{OLS}}$. Thereby, the constraint in problem (12) is equivalent to

(13) $$\begin{array}{cc} \; & E_{{\hat{P}}_{N}\left(\varepsilon^{\mathrm{OLS}}\right)}E_{\gamma_{\varepsilon^{\mathrm{OLS}}}\left(\varepsilon^{\prime }\right)}\left[|\varepsilon^{\mathrm{OLS}}-\varepsilon^{\prime }{|}^{p}+\eta \log\left(\frac{d\gamma_{\varepsilon^{\mathrm{OLS}}}\left(\varepsilon^{\prime }\right)}{d\varepsilon^{\prime }}\right)\right]\leq r. \end{array}$$

Note that any feasible solution γ satisfies $\gamma \ll {\hat{P}}_{N}\left(\varepsilon^{\mathrm{OLS}}\right)⊗\nu$, and hence, $\gamma_{\varepsilon^{\mathrm{OLS}}}\ll \nu$. Since ν is the Lebesgue measure in R, the term $\log\left(\frac{d\gamma_{\varepsilon^{\mathrm{OLS}}}\left(\varepsilon^{\prime }\right)}{d\varepsilon^{\prime }}\right)$ is well-defined. By applying the change-in-measure identity, we have

$$\log\left(\frac{d\gamma_{\varepsilon^{\mathrm{OLS}}}\left(\varepsilon^{\prime }\right)}{d\varepsilon^{\prime }}\right)=\log\left(\frac{dQ_{\varepsilon^{\mathrm{OLS}},\eta }\left(\varepsilon^{\prime }\right)}{d\varepsilon^{\prime }}\right)+\log\left(\frac{d\gamma_{\varepsilon^{\mathrm{OLS}}}\left(\varepsilon^{\prime }\right)}{dQ_{\varepsilon^{\mathrm{OLS}},\eta }\left(\varepsilon^{\prime }\right)}\right),$$

in which

$$\frac{dQ_{\varepsilon^{\mathrm{OLS}},\eta }\left(\varepsilon^{\prime }\right)}{d\varepsilon^{\prime }}=\frac{e^{-|\varepsilon^{\mathrm{OLS}}-\varepsilon^{\prime }{|}^{p}/\eta }}{\int_{R}e^{-|\varepsilon^{\mathrm{OLS}}{-\zeta |}^{p}/\eta }d\zeta }.$$

Thus, constraint (13) can be reformulated as

(14) $$E_{{\hat{P}}_{N}\left(\varepsilon^{\mathrm{OLS}}\right)}E_{\gamma_{\varepsilon^{\mathrm{OLS}}}\left(\varepsilon^{\prime }\right)}\left[|\varepsilon^{\mathrm{OLS}}-\varepsilon^{\prime }{|}^{p}+\eta \log\left(\frac{e^{-|\varepsilon^{\mathrm{OLS}}-\varepsilon^{\prime }{|}^{p}/\eta }}{\int_{R}e^{-|\varepsilon^{\mathrm{OLS}}{-\zeta |}^{p}/\eta }d\zeta }\right)+\eta \log\left(\frac{d\gamma_{\varepsilon^{\mathrm{OLS}}}\left(\varepsilon^{\prime }\right)}{dQ_{\varepsilon^{\mathrm{OLS}},\eta }\left(\varepsilon^{\prime }\right)}\right)\right]\leq r,$$

where $\gamma_{\varepsilon^{\mathrm{OLS}}}\left(\varepsilon^{\prime }\right)\in P\left(R\right)$. For the integrand in the expectation term on the left-hand side of constraint (14), we can write

$$\eta \log\left(\frac{e^{-|\varepsilon^{\mathrm{OLS}}-\varepsilon^{\prime }{|}^{p}/\eta }}{\int_{R}e^{-|\varepsilon^{\mathrm{OLS}}{-\zeta |}^{p}/\eta }d\zeta }\right)=-{\left|\varepsilon^{\mathrm{OLS}}-\varepsilon^{\prime }\right|}^{p}-\eta \log\left(\int_{R}e^{-|\varepsilon^{\mathrm{OLS}}{-\zeta |}^{p}/\eta }d\zeta \right).$$

By combining it with the first term $|\varepsilon^{\mathrm{OLS}}-\varepsilon^{\prime }{|}^{p}$, we have (14) being equivalent to

(15) $$\eta E_{{\hat{P}}_{N}\left(\varepsilon^{\mathrm{OLS}}\right)}E_{\gamma_{\varepsilon^{\mathrm{OLS}}}\left(\varepsilon^{\prime }\right)}\left[\log\left(\frac{d\gamma_{\varepsilon^{\mathrm{OLS}}}\left(\varepsilon^{\prime }\right)}{dQ_{\varepsilon^{\mathrm{OLS}},\eta }\left(\varepsilon^{\prime }\right)}\right)\right]\leq \bar{r}.$$

Similarly, the objective function of problem (10) can be written as

$$E_{{\hat{P}}_{N}\left(\varepsilon^{\mathrm{OLS}}\right)}E_{\gamma_{\varepsilon^{\mathrm{OLS}}}\left(\varepsilon^{\prime }\right)}\left[\rho_{\alpha }\left(\varepsilon^{\prime }-s\right)\right].$$

By introducing the Lagrange multiplier λ associated with constraint (15), we can reformulate problem (10) as

(16) $$\underset{\gamma_{\varepsilon^{\mathrm{OLS}}\left(\varepsilon^{\prime }\right)}\in P\left(R\right)}{\sup}\;\underset{\lambda \geq 0}{\inf}\left\{E_{{\hat{P}}_{N}\left(\varepsilon^{\mathrm{OLS}}\right)}E_{\gamma_{\varepsilon^{\mathrm{OLS}}}\left(\varepsilon^{\prime }\right)}\left[\rho_{\alpha }\left(\varepsilon^{\prime }-s\right)-\lambda \eta \log\left(\frac{d\gamma_{\varepsilon^{\mathrm{OLS}}}\left(\varepsilon^{\prime }\right)}{dQ_{\varepsilon^{\mathrm{OLS}},\eta }\left(\varepsilon^{\prime }\right)}\right)\right]+\lambda \bar{r}\right\}.$$

Note that its objective function $\rho_{\alpha }\left(\varepsilon^{\prime }-s\right)$ is convex in $\varepsilon^{\prime }$, and the feasible region $B_{r,\eta }\left({\hat{P}}_{N}\left(\varepsilon^{\mathrm{OLS}}\right)\right)$ is a convex set; we have that problem (10) is a convex problem, and thus, the strong duality holds. Therefore, under Assumption 1, according to Theorem 1 of [27], problem (16) is equivalent to

(17) $$\begin{array}{cc} \; & \underset{\lambda \geq 0}{\inf}\;\underset{\gamma_{\varepsilon^{\mathrm{OLS}}}\left(\varepsilon^{\prime }\right)\in P\left(R\right)}{\sup}\left\{E_{{\hat{P}}_{N}\left(\varepsilon^{\mathrm{OLS}}\right)}E_{\gamma_{\varepsilon^{\mathrm{OLS}}}\left(\varepsilon^{\prime }\right)}\left[\rho_{\alpha }\left(\varepsilon^{\prime }-s\right)-\lambda \eta \log\left(\frac{d\gamma_{\varepsilon^{\mathrm{OLS}}}\left(\varepsilon^{\prime }\right)}{dQ_{\varepsilon^{\mathrm{OLS}},\eta }\left(\varepsilon^{\prime }\right)}\right)\right]+\lambda \bar{r}\right\}\\ = & \underset{\lambda \geq 0}{\inf}\left\{\lambda \bar{r}+\lambda \eta E_{{\hat{P}}_{N}\left(\varepsilon^{\mathrm{OLS}}\right)}\left[\log\;E_{Q_{\varepsilon^{\mathrm{OLS}},\eta }\left(\varepsilon^{\prime }\right)}\left(e^{\rho_{\alpha }\left(\varepsilon^{\prime }-s\right)/\left(\lambda \eta \right)}\right)\right]\right\}. \end{array}$$

For i=1,⋯,N, given $\varepsilon^{\mathrm{OLS}}=\varepsilon_{i}^{\mathrm{OLS}}$, denote $Q_{i}:=Q_{\varepsilon^{\mathrm{OLS}},\eta }$, i∈[N]. One can verify that for any $\left(\varepsilon^{\mathrm{OLS}},\varepsilon^{\prime }\right)$, there exist distributions ${\left\{Q_{i}\right\}}_{i=1}^{N}$ such that the joint distribution $\gamma :=\frac{1}{N}\sum_{i=1}^{N}\delta_{\varepsilon_{i}^{\mathrm{OLS}}}⊗Q_{i}$. As a result, problem (17) is equivalent to

(18) $$\underset{\lambda \geq 0}{\inf}\left\{\lambda \bar{r}+\frac{\lambda \eta }{N}\sum_{i=1}^{N}\log\left(E_{Q_{i}\left(\varepsilon^{\prime }\right)}\left[e^{\rho_{\alpha }\left(\varepsilon^{\prime }-s\right)/\left(\lambda \eta \right)}\right]\right)\right\}.$$

Therefore, problem (6) is equivalent to problem (8). This completes the proof. □

From a computational perspective, the dual reformulation of Sinkhorn DRCQP (8) is non-trivial, as it can be viewed as a conditional stochastic optimization problem [36]. To solve it efficiently, ref. [27] proposed an efficient static mirror descent algorithm with biased subgradient estimators for Sinkhorn DRO problems, and ref. [40] developed a Nested-SGD algorithm designed for large-scale regularized Sinkhorn DRO problems. Detailed discussions of sample complexity and convergence guarantees are provided in these works.

Following Wang et al. [27], to distinguish the two cases F(s,λ)<∞ and F(s,λ)=∞, we introduce the light-tail condition on the quantile loss function $\rho_{\alpha }\left(\varepsilon^{\prime }-s\right)$.

**Assumption** **2.** *$\tilde{\lambda }>0$ exists such that $E_{Q_{i}\left(\varepsilon^{\prime }\right)}\left[e^{|\varepsilon^{\prime }-s|/\left(\tilde{\lambda }\eta \right)}\right]<\infty$ for ${\hat{P}}_{N}$ almost every $\varepsilon^{\mathrm{OLS}}$.*

Moreover, when the support Z is finite, the dual formulation (8) can be reformulated as the following conic programming problem under Assumption 2 (above). Therefore, the new formulation can be solved efficiently using off-the-shelf solvers such as CVX [41], which employ the interior point method.

**Theorem** **2** (Conic reformulation for finite support)**.** *Suppose that the support Z contains $L_{\max}$ elements, i.e., $Z={\left\{\varepsilon_{j}^{\prime }\right\}}_{j=1}^{L_{\max}}$. If Assumption 2 holds and $\bar{r}\geq 0$, problem* (8) *can be formulated as the following conic optimization:*

(19) $$\begin{array}{cc} \min_{\begin{array}{c} \lambda ,s,g,a \end{array}} & \;\lambda \bar{r}+\frac{1}{N}\sum_{i=1}^{N}g_{i}\\ s.t. & \;\lambda \eta \geq \sum_{j=1}^{L_{\max}}q_{i,j}a_{i,j},\;i\in \left[N\right],\\ \; & \;\left(\lambda \eta ,a_{i,j},\rho_{\alpha }\left(\varepsilon_{j}^{\prime }-s\right)-g_{i}\right)\in K_{\exp},\;\;i\in \left[N\right],\;j\in \left[L_{\max}\right],\\ \; & \;\lambda \geq 0,\;s\in R,\;g\in R^{N},\;a\in R^{N\times L_{\max}}. \end{array}$$

*where $q_{i,j}:=P_{Q_{i}\left(\varepsilon^{\prime }\right)}\left\{\varepsilon^{\prime }=\varepsilon_{j}^{\prime }\right\}$ with the distribution $Q_{i}\left(\varepsilon^{\prime }\right)$ defined in* (9)*, and $K_{\exp}$ denotes the exponential cone $K_{\exp}=\left\{\left(\nu ,\lambda ,\delta \right)\in R_{+}\times R_{+}\times R:\;\exp\left(\delta /\nu \right)\leq \lambda /\nu \right\}$.*

**Proof of Theorem** **2.** We now introduce the epi-graphical variables $g_{i},i=1,\cdots ,N$ to reformulate the dual objective (8) as

$$\begin{array}{cc} \min_{s,\lambda ,g_{i}} & \;\lambda \bar{r}+\frac{1}{N}\sum_{i=1}^{N}g_{i}\\ s.t. & \;\lambda \eta \log\left(E_{Q_{i}\left(\varepsilon^{\prime }\right)}\left[e^{\rho_{\alpha }\left(\varepsilon^{\prime }-s\right)/\left(\lambda \eta \right)}\right]\right)\leq g_{i},\;\forall i\\ \; & \;s\in R,\;\lambda >0,\;g_{i}\in R. \end{array}$$

For a fixed *i*, the *i*-th constraint can be reformulated as

$$\begin{array}{cc} \; & \left\{\exp\left(\frac{g_{i}}{\lambda \eta }\right)\geq E_{Q_{i}\left(\varepsilon^{\prime }\right)}\left[e^{\rho_{\alpha }\left(\varepsilon^{\prime }-s\right)/\left(\lambda \eta \right)}\right]\right\}\\ = & \left\{1\geq E_{Q_{i}\left(\varepsilon^{\prime }\right)}\left[e^{\left(\rho_{\alpha }\left(\varepsilon^{\prime }-s\right)-g_{i}\right)/\left(\lambda \eta \right)}\right]\right\}\\ = & \left\{\lambda \eta \geq E_{Q_{i}\left(\varepsilon^{\prime }\right)}\left[\lambda \eta e^{\left(\rho_{\alpha }\left(\varepsilon^{\prime }-s\right)-g_{i}\right)/\left(\lambda \eta \right)}\right]\right\}\\ = & \left\{\lambda \eta \geq \sum_{j=1}^{L_{\max}}q_{i,j}a_{i,j}\right\}⋂\left\{a_{i,j}\geq \lambda \eta \exp\left(\frac{\rho_{\alpha }\left(\varepsilon_{j}^{\prime }-s\right)-g_{i}}{\lambda \eta }\right),\forall j\right\}, \end{array}$$

where the second constraint set can be formulated as

$$\left(\lambda \eta ,a_{i,j},\rho_{\alpha }\left(\varepsilon_{j}^{\prime }-s\right)-g_{i}\right)\in K_{\exp}.$$

Substituting this expression into the dual objective completes the proof. □

The following remarks elucidate the connection between the Sinkhorn DRCQP problem (8) and the DRCQP problems based on other ambiguity sets.

**Remark** **1** (Connection with Wasserstein DRCQP)**.** *As the regularization parameter η→0, formulation* (6) *reduces to*

$$\min_{s}\;\underset{P_{\varepsilon^{\prime }}\in P\left(\varepsilon^{\prime }\right)}{\sup}E_{P_{\varepsilon^{\prime }}}\left[\rho_{\alpha }\left(\varepsilon^{\prime }-s\right)\right],$$

*where $P\left(\varepsilon^{\prime }\right)=\left\{P_{\varepsilon^{\prime }}:W{\left({\hat{P}}_{N}\left(\varepsilon^{\mathrm{OLS}}\right),P_{\varepsilon^{\prime }}\right)}^{p}\leq r\right\}$ is the ambiguity set with Wasserstein distance $W{\left(P,Q\right)}^{p}=\underset{\gamma \in \Gamma (P,Q)}{\inf}\left\{E_{(z,z^{\prime })∼\gamma }\left[d{\left(z,z^{\prime }\right)}^{p}\right]\right\}$. Furthermore, the dual objective of the Sinkhorn DRCQP problem* (8) *converges to*

$$\lambda r+\frac{1}{N}\sum_{i=1}^{N}\left[\underset{\varepsilon^{\prime }\in R}{\sup}\left\{\rho_{\alpha }\left(\varepsilon^{\prime }-s\right)-\lambda {\left|\varepsilon_{i}^{\mathrm{OLS}}-\varepsilon^{\prime }\right|}^{p}\right\}\right].$$

*For the complete proof, we refer to Remark 3 in [27]. The derivation and simplified results for the cases p=1 and p>1 are provided in [9] and [37], respectively.*

**Remark** **2** (Connection with KL DRCQP)**.** *Applying Jensen’s inequality, we obtain an upper bound for the dual objective function of the Sinkhorn DRCQP*

(20) $$\lambda \bar{r}+\lambda \eta \log\left(\frac{1}{N}\sum_{i=1}^{N}E_{Q_{i}\left(\varepsilon^{\prime }\right)}\left[e^{\rho_{\alpha }\left(\varepsilon^{\prime }-s\right)/\left(\lambda \eta \right)}\right]\right),$$

*which corresponds to the dual objective for the following KL DRO [19]*

(21) $$\min_{s}\;\underset{P_{\varepsilon^{\prime }}\in P\left(\varepsilon^{\prime }\right)}{\sup}E_{P_{\varepsilon^{\prime }}}\left[\rho_{\alpha }\left(\varepsilon^{\prime }-s\right)\right],$$

*where $P\left(\varepsilon^{\prime }\right)=\left\{P_{\varepsilon^{\prime }}:D_{KL}\left(P_{\varepsilon^{\prime }}∥P^{0}\right)\leq \bar{r}/\eta \right\}$ is the ambiguity set with KL divergence $D_{KL}\left(P_{\varepsilon^{\prime }}∥P^{0}\right)=E_{P_{\varepsilon^{\prime }}}\left[\log\left(\frac{dP_{\varepsilon^{\prime }}\left(\varepsilon^{\prime }\right)}{dP^{0}\left(\varepsilon^{\prime }\right)}\right)\right]$, and $P^{0}$ satisfies $dP^{0}\left(\varepsilon^{\prime }\right)=\frac{1}{N}\sum_{i=1}^{N}\left[dQ_{i}\left(\varepsilon^{\prime }\right)\right]$.*

**Proof of Remark** **2.** Recall that Assumption 2 requires that the quantile loss $\rho_{\alpha }\left(\varepsilon^{\prime }-s\right)$ has a light tail under the distribution $Q_{i}\left(\varepsilon^{\prime }\right)$ for ${\hat{P}}_{N}$ almost every $\varepsilon^{\mathrm{OLS}}$, which satisfies Assumption 1 in [18]. Consequently, by applying Theorem 1 in [18], problem (21) is equivalent to the following one-layer optimization problem:

(22) $$\begin{array}{c} \min_{\lambda^{\prime }\geq 0}\left\{\frac{\lambda^{\prime }\bar{r}}{\eta }+\lambda^{\prime }\log\left(E_{P^{0}\left(\varepsilon^{\prime }\right)}\left[e^{\rho_{\alpha }\left(\varepsilon^{\prime }-s\right)/\lambda^{\prime }}\right]\right)\right\}. \end{array}$$

Substituting $\lambda =\frac{\lambda^{\prime }}{\eta }$ and $dP^{0}\left(\varepsilon^{\prime }\right)=\frac{1}{N}\sum_{i=1}^{N}\left[dQ_{i}\left(\varepsilon^{\prime }\right)\right]$, we can obtain problem (20). This completes the proof. □

## 5. Numerical Experiments

In Section 5.1, our numerical experiments on the simulated dataset are presented, and the real-world dataset is addressed in Section 5.2. First, for the conditional quantile prediction problem under the fixed design setting, we compare the out-of-sample performance and computational time of the Sinkhorn DRO method used in this paper with the SAA method [8], other DRO methods (such as Wasserstein DRO [9,37], KL DRO [19]), and other machine learning techniques, like robust quantile regression with Catoni’s log-truncated loss [42,43] and Cauchy-truncated loss [44]. Second, we conduct experiments to examine the impact of varying the entropy regularization parameter η on the out-of-sample performance of our Sinkhorn DRCQP problem.

In this paper, we adopt two metrics to evaluate the out-of-sample performance of all models. The first metric is the out-of-sample expected cost achieved by the optimal solution ${\hat{s}}_{N}$ to the conditional quantile prediction problem, obtained via the SAA method, the DRO method, and the machine learning methods, denoted as $J_{saa}$, $J_{dro}$, and $J_{ml}$, respectively. Additionally, for different DRO methods, their corresponding out-of-sample expected costs are denoted as $J_{{sink}_{p}}$ (Sinkhorn DRO), $J_{{wass}_{p}}$ (Wasserstein DRO), and $J_{kl}$ (KL DRO), where *p* represents the order of the cost function in the Sinkhorn distance and Wasserstein distance. For different machine learning methods, their corresponding out-of-sample expected costs are denoted as $J_{cat}$ (Catoni’s log-truncated robust quantile regression) and $J_{cau}$ (Cauchy-truncated robust quantile regression), respectively. Furthermore, the optimal solution ${\hat{s}}_{N}$ for each model can be expressed as follows.

- Conditional quantile prediction problem based on the SAA method [8]:
  $${\hat{\beta }}_{N},{\hat{s}}_{N}=\underset{\beta ,s}{\arg\min}\;\frac{1}{N}\sum_{i=1}^{N}\rho_{\alpha }\left(y_{i}-\beta^{⊤}x_{i}-s\right).$$
- KL DRCQP problem [19]:
  $${\hat{s}}_{N},{\hat{\lambda }}_{N}=\underset{s,\lambda }{\arg\min}\;\lambda \log\frac{1}{N}\sum_{i=1}^{N}\left[e^{\rho_{\alpha }\left(\varepsilon_{i}^{\mathrm{OLS}}-s\right)/\lambda }\right]+\lambda r.$$
- Type 1-Wasserstein DRCQP problem [9]:
  $${\hat{s}}_{N},{\hat{\lambda }}_{N}=\underset{s,\;\lambda \geq \max\{\alpha ,1-\alpha \}}{\arg\min}\;\lambda r+\frac{1}{N}\sum_{i=1}^{N}\rho_{\alpha }\left(\varepsilon_{i}^{\mathrm{OLS}}-s\right).$$
- Type *p*-Wasserstein (p≥2) DRCQP problem [37]:
  ${\hat{s}}_{N},{\hat{\lambda }}_{N}=\underset{s,\;\lambda \geq 0}{\arg\min}\;\lambda r+\frac{1}{N}\sum_{i=1}^{N}\rho_{\alpha }^{*}\left(\varepsilon_{i}^{\mathrm{OLS}}-s\right)+\lambda^{-\frac{1}{p-1}}\left(p-1\right)\left[{\left(\frac{\alpha }{p}\right)}^{\frac{p}{p-1}}I_{1}+{\left(\frac{1-\alpha }{p}\right)}^{\frac{p}{p-1}}I_{2}\right]$
  where $I_{1}=\frac{1}{N}\sum_{i=1}^{N}1_{\left\{\varepsilon_{i}^{\mathrm{OLS}}\geq s+a\right\}}$, $I_{2}=1-I_{1}$, $a={\left(\lambda p\right)}^{-\frac{1}{p-1}}\left(1-\frac{1}{p}\right)\left[{\left(1-\alpha \right)}^{\frac{p}{p-1}}-\alpha^{\frac{p}{p-1}}\right]$
  and $\rho_{\alpha }^{*}\left(u\right)=u\left(\alpha -1_{\left(u\leq a\right)}\right)=\left\{\begin{array}{cc} \alpha u & \mathrm{if}\;u>a,\\ (\alpha -1)u & \mathrm{if}\;u\leq a. \end{array}\right.$
- Catoni’s log-truncated robust quantile regression [43]:
  $${\hat{\beta }}_{N},{\hat{s}}_{N}=\underset{\beta ,s}{\arg\min}\;\frac{1}{N\kappa }\sum_{i=1}^{N}\Psi \left[\kappa \rho_{\alpha }\left(y_{i}-\beta^{⊤}x_{i}-s\right)\right],$$
  where κ>0 is a robustification parameter to be tuned, and the non-decreasing influence function Ψ:R→R is
  $$\Psi \left(u\right)=\left\{\begin{array}{cc} \log(1+u+\frac{u^{2}}{2}) & \mathrm{if}\;u>0,\\ -\log(1-u+\frac{u^{2}}{2}) & \mathrm{if}\;u\leq 0. \end{array}\right.$$
- Cauchy-truncated robust quantile regression [44]:
  $${\hat{\beta }}_{N},{\hat{s}}_{N}=\underset{\beta ,s}{\arg\min}\;\frac{1}{N}\sum_{i=1}^{N}\Phi \left[\rho_{\alpha }\left(y_{i}-\beta^{⊤}x_{i}-s\right)\right],$$
  where κ>0, and the truncation function Φ:R→R is $\Phi_{\kappa }\left(u\right)=\kappa \log\left(1+\frac{u}{\kappa }\right)$.

The second metric is the improvement achieved by the DRO strategy compared to the SAA approach, calculated as $\frac{J_{dro}-J_{saa}}{J_{saa}}$ [9].

### 5.1. Simulation

In this section, we evaluate the performance of our proposed method on the simulated dataset. We generate the dataset under the fixed design setting, following a similar setup to [45]. Define $m_{i}:\left[0,1\right]\to R$, i=1,2,3 as follows:

$$m_{1}\left(x\right)=\sin\left(5x\right),\;m_{2}\left(x\right)=e^{5x}-{\left(5x\right)}^{3},\;\mathrm{and}\;m_{3}\left(x\right)=\frac{1}{5x+1}+\sin\left(5x\right).$$

and the response variable is generated according to the model

$$y_{i}=m_{1}\left(x_{i}\right)+2m_{2}\left(x_{i}\right)+3m_{3}\left(x_{i}\right)+\varepsilon_{i},\;i=1,2,\ldots ,N,$$

where $x_{i}=i/n$, β=(1,2,3) and $\varepsilon_{i}$ are i.i.d. samples drawn from a heavy-tailed distribution (e.g., Pareto distribution and Student’s t-distribution). According to the above model, a total of 2000 groups of sample data were generated. The first 1500 groups of samples were used as the training set, and retraining was performed every 50 data points, with a total of 30 training sessions. The last 500 groups of samples served as the test set.

Our simulation experiment consists of two parts: Section 5.1.1 presents a comprehensive performance comparison between the Sinkhorn DRCQP problem and the other five problems. The comparison includes the out-of-sample expected cost and the computational time for solving each problem. Section 5.1.2 evaluates the impact of the entropy regularization parameter η on the final results of the Sinkhorn DRO.

#### 5.1.1. Comparison of Out-of-Sample Performance and Computational Time

**Figure 1:**
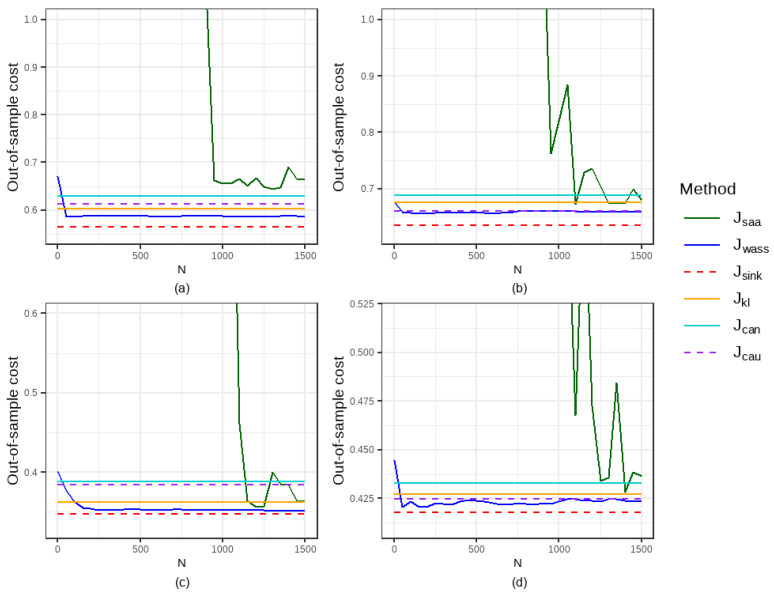
Out-of-sample performance of six methods under varying configurations with Student’s t-distributed ε: (**a**) α=0.2, p=1, η=0.01, and ε∼t(2); (**b**) α=0.7, p=2, η=0.05, and ε∼t(2); (**c**) α=0.2, p=1, η=0.05, and ε∼t(5); (**d**) α=0.7, p=2, η=0.05, and ε∼t(5).

**Figure 2:**
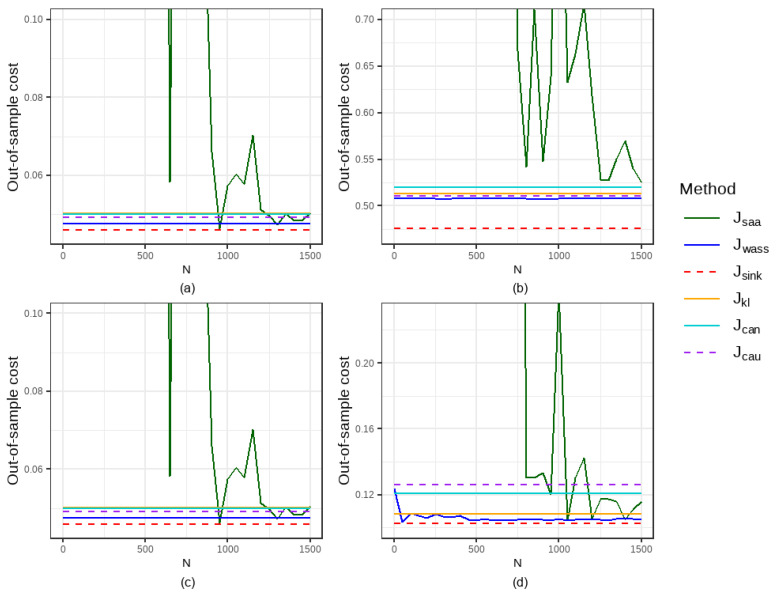
Out-of-sample performance of six methods under varying configurations with Pareto distribution ε: (**a**) α=0.2, p=1, η=0.05, and ε∼P(2,1); (**b**) α=0.7, p=2, η=0.05, and ε∼P(2,1); (**c**) α=0.2, p=1, η=0.01, and ε∼P(5,1); (**d**) α=0.7, p=2, η=0.4, and ε∼P(5,1).

In this section, we compare the out-of-sample performance of six methods on the simulated dataset under different configurations of the distribution of ε, the quantile α, the order of the cost function *p*, and the entropy regularization parameter η, with a fixed radius of the ambiguity set r=0.2. Figure 1 and Figure 2 illustrate the out-of-sample performance at intervals of 50 data points. Table 1 and Table 2 present the comparative out-of-sample performance and the computational time of six methods for the training sample sizes N=100, 800, and 1500, respectively.

Our results show that the proposed method in this paper outperforms the five traditional methods in most cases. As the sample size used for training increases, the out-of-sample expected costs of the SAA method gradually converge, while the out-of-sample costs of the DRO methods remain consistently stable and exhibit excellent performance. Although the out-of-sample performance of the two machine learning methods (denoted as $J_{cat}$ and $J_{cau}$; κ=1) is stable, their out-of-sample expected costs are higher than those of the DRO method in most cases. Additionally, the performance improvement of the DRO methods over the SAA method decreases as the training sample size expands. Among the three DRO methods, Sinkhorn DRO demonstrates the most remarkable performance improvement over the SAA method, which can also be significantly compared. For example, when taking ε∼P(2,1), α=0.7 and N=1500, the improvement in $J_{sink_{1}}$ over $J_{saa}$ is 9.40%, whereas the improvement of $J_{wass_{1}}$ over $J_{saa}$ is only 3.33%.

In Table 2, the total computation time required for each method is recorded as the sample size increases from 1 to 1500 at intervals of 50 data points. As shown in Table 2, although the Sinkhorn DRO ranks second in terms of the total computation time among the six methods, its out-of-sample performance is the best in most cases. Therefore, it combines computational efficiency and prediction accuracy. In contrast, while the KL DRO has a shorter computation time, its out-of-sample performance is inferior to that of the Sinkhorn DRO method. Meanwhile, although the Wasserstein DRO delivers comparable predictive accuracy to Sinkhorn DRO, its computational time ranges from 14 to 53 times longer, rendering it less efficient computationally. Notably, the SAA method shows both the poorest predictive performance and the lowest computational efficiency among all methods in most cases, indicating that it is not well-suited for solving conditional quantile prediction problems under the fixed design settings.

#### 5.1.2. Comparison of the Impacts of Parameter Settings

In this section, we analyze the effect of the entropy regularization parameter η on the out-of-sample performance of the Sinkhorn DRO method under the fixed design settings.

**Figure 3:**
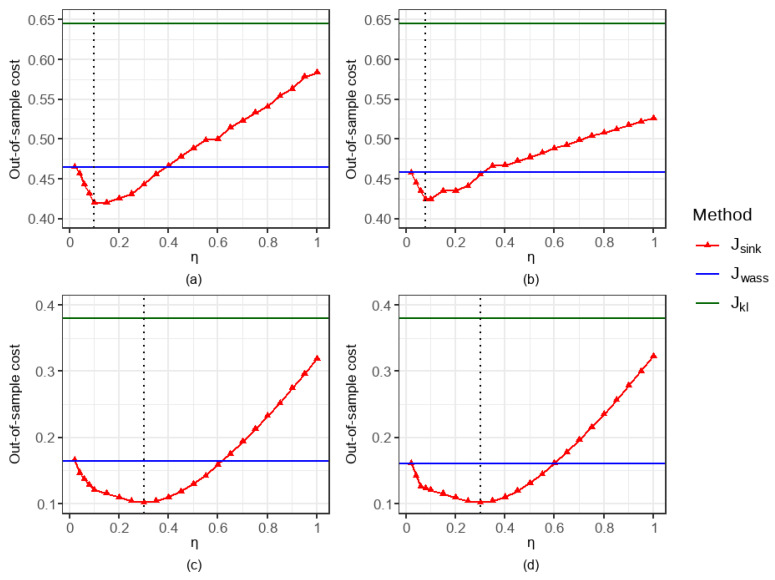
Out-of-sample performance of three DRO methods for α=0.7 and r=0.2 under varying configurations: (**a**) p=1 and ε∼t(5); (**b**) p=2 and ε∼t(5); (**c**) p=1 and ε∼P(5,1); (**d**) p=2 and ε∼P(5,1).

As shown in Figure 3, regardless of whether ε follows a heavy-tailed distribution (e.g., t(5) or P(5,1)), the Sinkhorn DRO method can achieve better predictive performance than both Wasserstein DRO and KL DRO through scientific adjustment of the entropy regularization parameter η. Specifically, when ε∼t(5), $J_{sink_{1}}$ reaches its minimum value of 0.4199 at η=0.1, while $J_{sink_{2}}$ achieves its minimum of 0.4243 at η=0.08. When ε∼P(5,1), both $J_{sink_{1}}$ and $J_{sink_{2}}$ attain their respective minimum of 0.1025 and 0.1023 at η=0.3. Furthermore, when η→0, the results of $J_{sink}$ approximate those of $J_{wass}$, which aligns with the theoretical results in Remark 1. As η continues to increase, $J_{sink}$ first reaches its optimal value before gradually deteriorating, though it still maintains a noticeable advantage over $J_{kl}$.

### 5.2. Real-World Applications

In this section, we validate the performance of our proposed method using the real-world dataset. Our goal was to predict the total demand for public bicycles in Seoul, South Korea, per hour, taking into account external factors such as temperature and humidity. By predicting the quantiles of the stochastic demand process, we can better manage bicycle inventory, ensure a stable supply of rental bicycles, and improve the comfort of public transportation. Section 5.2.1 provides an overview of the real-world dataset and its reconstruction steps. In Section 5.2.2, we evaluate the out-of-sample performance of Sinkhorn DRO against the following: (i) the SAA baseline, (ii) two DRO benchmarks (Wasserstein DRO and KL DRO), and (iii) two robust quantile regression methods (with Catoni’s log-truncated loss and Cauchy-truncated loss). In addition, Section 5.2.3 evaluates the impact of relevant parameters on the final results, providing guidance for effective parameter tuning.

#### 5.2.1. Data Selection and Reconstruction

The data used in the real-world applications were sourced from the UCI Machine Learning Repository, established in [46]. It includes a real dataset of the hourly rental count of public bicycles in Seoul, South Korea (which serves as the response variable, denoted as *Y*), along with corresponding weather data and information about holidays. In total, there are 8760 valid instances (valid means that the hourly rental count of public bicycles is not zero). To account for the influence of multicollinearity, we ultimately retained seven covariates to construct the design matrix $X^{f}$. The specific information about these covariates is shown in Table 3.

Since the observations are time series data, and covariates, such as temperature, humidity, and wind speed, tend to be closely correlated within a few consecutive days, the observations should not be treated as i.i.d. data. This means we need to consider the fixed design setting. The specific steps we take to process the dataset are as follows.

- Step 1: Construct a linear regression problem on the preprocessed dataset and use the coefficients estimated using the OLS method as the coefficients for potential true linear relationships, namely $\beta_{0}$;
- Step 2: Discard the demand column of the dataset and retain only the columns of the covariates. Then, generate new demand observations $y_{i}$ by adding i.i.d. simulated noise $\varepsilon_{i}$ following the normal distribution. Specifically, $y_{i}={\beta_{0}}^{⊤}x_{i}+\varepsilon_{i};$
- Step 3: Divide the observations into training and testing sets based on time periods. Starting from the 8000-th observation, the next 500 time periods are used as the test set. For any given sample size *N*, the training set consists of the *N* observations immediately preceding the start of the test set;
- Step 4: For each observation in the test set, i.i.d. noises are simulated 50 times, resulting in a total of 500×50=25,000 data points for the test set. These data points are used to evaluate the predictive performance of each method.

#### 5.2.2. Comparison of Out-of-Sample Performance and Computational Time

**Figure 4:**
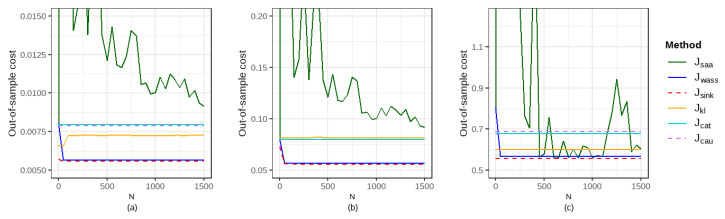
Out-of-sample performance of six methods for α=0.2 and p=1 under varying σ and η: (**a**) σ=0.02,η=0.005; (**b**) σ=0.2,η=0.01; (**c**) σ=2,η=0.8.

**Figure 5:**
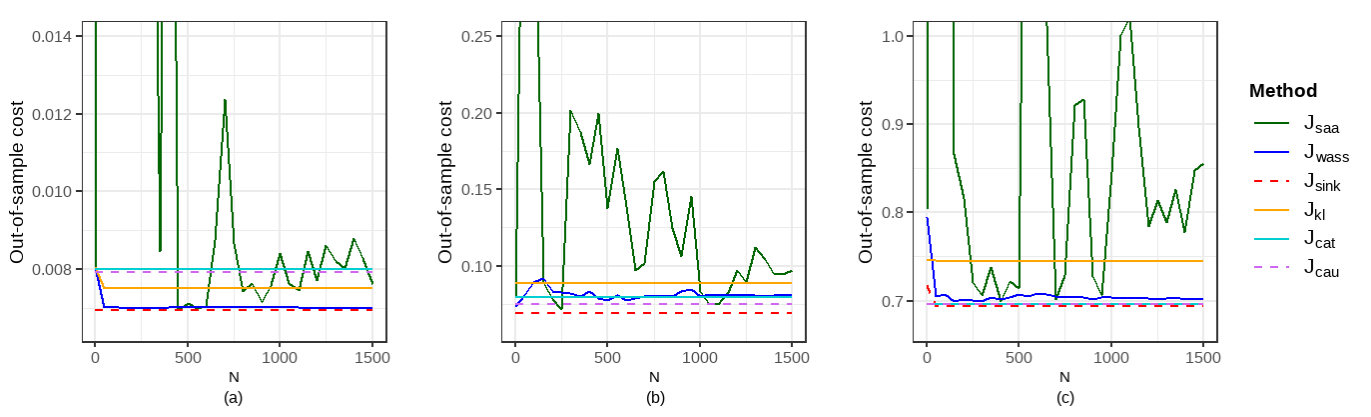
Out-of-sample performance of six methods for α=0.7 and p=2 under varying σ and η: (**a**) σ=0.02, η=1; (**b**) σ=0.2, η=0.05; (**c**) σ=2, η=0.5.

In this section, we compare the out-of-sample performance of six methods under different configurations of the quantile α, the standard deviation of the error term σ, the order of the cost function *p*, and the entropy regularization parameter η, with a fixed radius of the ambiguity set r=0.05. Figure 4 and Figure 5 illustrate the out-of-sample expected costs at intervals of 50 data points. Table 4 presents the comparative out-of-sample performance of the six methods for sample sizes N=100, 550, and 1500, with $\frac{J_{dro}-J_{saa}}{J_{saa}}$ shown in parentheses. Our results show that the proposed method outperforms the SAA method, two DRO benchmarks (Wasserstein DRO and KL DRO), and two robust quantile regression methods (with Catoni’s log-truncated loss and Cauchy-truncated loss) in most cases.

Specifically, we can observe that while the SAA method maintains some convergence for large samples, its out-of-sample performance is poor in the case of small samples. This is mainly because the data points in the training and testing sets are not randomly generated, failing to meet the assumption of i.i.d. conditions. Therefore, the performance guarantee of the SAA method under the fixed design setting is no longer applicable [9]. Moreover, while both Wasserstein DRO and KL DRO can be employed to formulate conditional quantile problems under the fixed design setting and demonstrate relatively stable performance, in most scenarios, their out-of-sample performance is slightly inferior to the Sinkhorn DRO method. In addition, compared to robust quantile regression using Catoni’s log-truncated loss and Cauchy-truncated loss when κ=1, it is found that in most cases, Sinkhorn DRO is outperformed by neither method, and both robust regression techniques are shown to outperform traditional quantile regression methods.

#### 5.2.3. Comparison of the Impacts of Parameter Settings

In this section, we analyze the effect of the entropy regularization parameter η on the out-of-sample performance of our Sinkhorn DRCQP problem.

**Figure 6:**
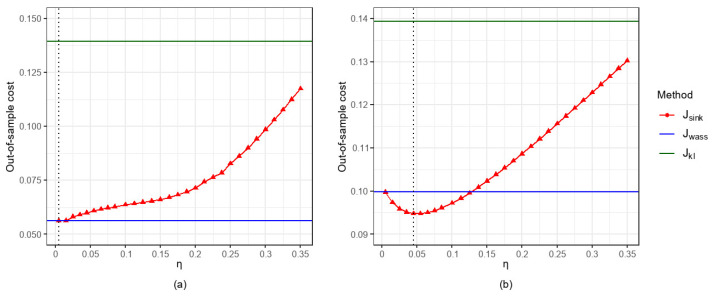
Out-of-sample performance for α=0.2,σ=0.2, N=500, and r=0.2: (**a**) p=1; (**b**) p=2.

**Figure 7:**
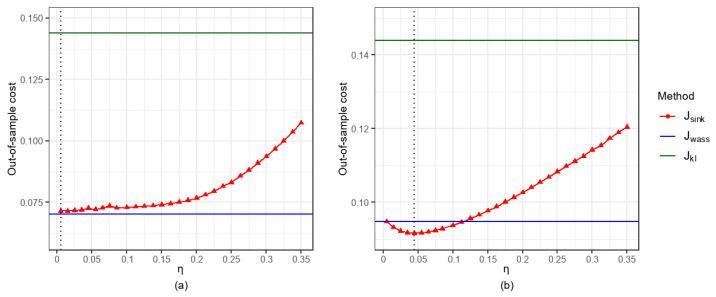
Out-of-sample performance for α=0.7,σ=0.2, N=500, and r=0.2: (**a**) p=1; (**b**) p=2.

Overall, for the fixed α,σ,N,p,r, we compute the corresponding out-of-sample expected cost $J_{sink}$ and compare it with $J_{wass}$ and $J_{kl}$ under the same settings. Figure 6a and Figure 7a show that when p=1, as η increases, $J_{sink}$ exhibits an increasing trend. Figure 6b and Figure 7b show that when p=2, as η increases, $J_{sink}$ first decreases and subsequently increases.

Specifically, for the case p=2, when η=0.005, the results of $J_{sink}$ (0.0997 in Figure 6b and 0.0947 in Figure 7b) are remarkably close to those of $J_{wass}$ (0.0999 in Figure 6b and 0.0948 in Figure 7b), which validates Remark 1 in this paper. As η increases to 0.045, $J_{sink}$ achieves its minimum values of 0.0948 in Figure 6b and 0.0916 in Figure 7b. When η>0.045, $J_{sink}$ deteriorates rapidly but consistently remains lower than $J_{kl}$ (0.1394 in Figure 6b and 0.1440 in Figure 7b). Generally speaking, Figure 6b and Figure 7b clearly demonstrate that by selecting appropriate values of η, the Sinkhorn DRCQP can outperform both the classic Wasserstein- and the KL divergence-based DRCQP approaches.

## 6. Conclusions

This paper proposes a Sinkhorn DRCQP problem under the fixed design setting. We demonstrate the tractability of the proposed model by deriving its strong dual formulation, which takes the form of an exponential cone programming problem for finite support cases. Finally, we conduct numerical experiments to evaluate the performance and computational time of the proposed method in comparison with five conventional methods, including SAA, Wasserstein DRO, KL DRO, Catoni’s log-truncated robust quantile regression, and Cauchy-truncated robust quantile regression. The experimental results demonstrate the superior performance of our approach by choosing suitable values of the entropy regularization parameter η.

## Figures and Tables

**Table 1:** Out-of-sample performance of six methods.

Noise	α	*N*	$J_{saa}$	$J_{cat}$	$J_{cau}$	$J_{{\mathrm{wass}}_{1}}$	$J_{{\mathrm{wass}}_{2}}$	$J_{{\mathrm{sink}}_{1}}$	$J_{{\mathrm{sink}}_{2}}$	$J_{\mathrm{kl}}$
t(2)	0.2	100	7.78e+04	0.630	0.612	0.574 (−99.99%)	0.574 (−99.99%)	0.559 (−99.99%)	0.559 (−99.99%)	0.603 (−99.99%)
		800	1.060	0.630	0.612	0.587 (−44.63%)	0.586 (−44.68%)	0.564 (−46.76%)	0.559 (−47.31%)	0.603 (−43.12%)
		1500	0.664	0.630	0.612	0.587 (−11.61%)	0.581 (−12.55%)	0.564 (−14.99%)	0.559 (−15.87%)	0.603 (−9.18%)
	0.7	100	1.75e+04	0.688	0.660	0.667 (−99.99%)	0.657 (−99.99%)	0.648 (−99.99%)	0.635 (−99.99%)	0.676 (−99.99%)
		800	3.842	0.688	0.660	0.664 (−82.71%)	0.661 (−82.80%)	0.648 (−83.14%)	0.635 (−83.48%)	0.676 (−82.41%)
		1500	0.680	0.688	0.660	0.665 (−2.23%)	0.659 (−3.14%)	0.648 (−4.75%)	0.635 (−6.65%)	0.676 (−0.62%)
t(5)	0.2	100	8.50e+03	0.389	0.384	0.363 (−99.99%)	0.371 (−99.99%)	0.348 (−99.99%)	0.348 (−99.99%)	0.362 (−99.99%)
		800	8.755	0.389	0.384	0.353 (−95.97%)	0.355 (−95.94%)	0.348 (−96.03%)	0.348 (−96.03%)	0.362 (−95.86%)
		1500	0.364	0.389	0.384	0.351 (−3.42%)	0.358 (−1.53%)	0.348 (−4.40%)	0.348 (−4.40%)	0.362 (−0.42%)
	0.7	100	2.86e+03	0.433	0.425	0.428 (−99.99%)	0.423 (−99.99%)	0.418 (−99.99%)	0.418 (−99.99%)	0.427 (−99.99%)
		800	6.885	0.433	0.425	0.421 (−93.88%)	0.422 (−93.87%)	0.418 (−93.93%)	0.418 (−93.93%)	0.427 (−93.79%)
		1500	0.436	0.433	0.425	0.421 (−3.59%)	0.424 (−2.94%)	0.418 (−4.33%)	0.418 (−4.31%)	0.427 (−2.09%)
P(2,1)	0.2	100	2.16e+03	0.196	0.194	0.193 (−99.99%)	0.193 (−99.99%)	0.183 (−99.99%)	0.183 (−99.99%)	0.194 (−99.99%)
		800	0.673	0.196	0.194	0.193 (−71.38%)	0.193 (−71.38%)	0.183 (−72.82%)	0.183 (−72.82%)	0.194 (−71.11%)
		1500	0.194	0.196	0.194	0.193 (−0.97%)	0.193 (−0.97%)	0.183 (−5.95%)	0.183 (−5.95%)	0.194 (−0.01%)
	0.7	100	4.38e+04	0.519	0.511	0.508 (−99.99%)	0.508 (−99.99%)	0.476 (−99.99%)	0.476 (−99.99%)	0.513 (−99.99%)
		800	0.542	0.519	0.511	0.508 (−6.32%)	0.508 (−6.31%)	0.476 (−12.20%)	0.476 (−12.20%)	0.513 (−5.33%)
		1500	0.525	0.519	0.511	0.508 (−3.33%)	0.508 (−3.31%)	0.476 (−9.40%)	0.476 (−9.40%)	0.513 (−2.30%)
P(5,1)	0.2	100	1.06e+03	0.050	0.049	0.047 (−99.99%)	0.049 (−99.99%)	0.046 (−99.99%)	0.046 (−99.99%)	0.050 (−99.99%)
		800	0.225	0.050	0.049	0.047 (−78.85%)	0.049 (−78.28%)	0.046 (−79.56%)	0.046 (−79.56%)	0.050 (−77.66%)
		1500	0.050	0.050	0.049	0.047 (−5.89%)	0.049 (−3.36%)	0.046 (−9.04%)	0.046 (−9.04%)	0.050 (−0.57%)
	0.7	100	1.43e+03	0.121	0.126	0.107 (−99.99%)	0.109 (−99.99%)	0.102 (−99.99%)	0.102 (−99.99%)	0.108 (−99.99%)
		800	0.130	0.121	0.126	0.107 (−17.92%)	0.105 (−19.66%)	0.102 (−21.46%)	0.102 (−21.53%)	0.108 (−16.95%)
		1500	0.116	0.121	0.126	0.107 (−7.60%)	0.105 (−9.62%)	0.102 (−11.58%)	0.102 (−11.67%)	0.108 (−6.51%)

Here, t(ζ) denotes the Student’s t-distribution with ζ degrees of freedom, and P(a,b) denotes the Pareto distribution with shape parameter *a* and scale parameter *b*. The values in parentheses in our tables are all calculated using the formula $\left(J_{\mathrm{dro}}-J_{\mathrm{saa}}\right)/J_{\mathrm{saa}}$.

**Table 2:** Total computational time (in seconds) of six methods.

Noise	α	SAA	CAT	CAU	1-WDRO	2-WDRO	1-SDRO	2-SDRO	KL-DRO
t(2)	0.2	2.11e+02	3.35e+02	5.39e+02	1.80e+02	1.05e+02	4.76e+00	3.23e+00	1.90e-01
	0.7	2.48e+02	3.17e+02	2.27e+02	1.53e+02	9.85e+01	5.39e+00	2.14e+00	2.00e-01
t(5)	0.2	4.18e+02	3.40e+02	5.13e+02	9.43e+01	8.89e+01	4.58e+00	2.12e+00	1.70e-01
	0.7	3.90e+02	3.35e+02	4.00e+02	9.96e+01	9.18e+01	4.75e+00	2.59e+00	1.90e-01
P(2,1)	0.2	3.26e+02	2.91e+02	3.16e+02	1.29e+02	1.38e+02	5.08e+00	3.17e+00	1.50e-01
	0.7	3.81e+02	1.99e+02	2.44e+02	9.82e+01	1.01e+02	4.34e+00	1.95e+00	2.00e-01
P(5,1)	0.2	2.88e+02	3.38e+02	5.05e+02	9.67e+01	1.03e+02	6.62e+00	2.37e+00	1.50e-01
	0.7	3.62e+02	3.62e+02	3.44e+02	8.92e+01	9.19e+01	5.85e+00	3.97e+00	1.70e-01

CAT, CAU, 1-WDRO, 2-WDRO, 1-SDRO, and 2-WDRO represent Catoni’s log-truncated robust quantile regression, Cauchy-truncated robust quantile regression, type-1 Wasserstein DRO, type-2 Wasserstein DRO, type-1 Sinkhorn DRO, and type-2 Sinkhorn DRO, respectively.

**Table 3:** Summary of covariates.

Feature	Type	Value Range	Unit
Temperature	Numeric	(−17.8, 39.4)	°C
Humidity	Numeric	(0, 98)	%
Wind speed	Numeric	(0, 7.4)	m/s
Visibility	Numeric	(270, 20000)	m
Dew point temperature	Numeric	(−30.6, 27.2)	°C
Solar radiation	Numeric	(0, 3.52)	MJ/m^2^
Rainfall	Numeric	(0, 35)	mm

**Table 4:** Out-of-sample performance of six methods.

σ	α	*N*	$J_{\mathrm{saa}}$	$J_{\mathrm{cat}}$	$J_{\mathrm{cau}}$	$J_{{\mathrm{wass}}_{1}}$	$J_{{\mathrm{wass}}_{2}}$	$J_{{\mathrm{sink}}_{1}}$	$J_{{\mathrm{sink}}_{2}}$	$J_{\mathrm{kl}}$
0.02	0.2	100	0.031	0.008	0.008	0.006 (−81.91%)	0.006 (−81.91%)	0.006 (−82.13%)	0.006 (−82.12%)	0.007 (−76.73%)
		550	0.014	0.008	0.008	0.006 (−60.53%)	0.006 (−60.53%)	0.006 (−60.99%)	0.006 (−60.97%)	0.007 (−49.20%)
		1500	0.009	0.008	0.008	0.006 (−38.39%)	0.006 (−38.39%)	0.006 (−39.11%)	0.006 (−39.07%)	0.007 (−20.71%)
	0.7	100	0.041	0.008	0.008	0.007 (−83.03%)	0.007 (−83.03%)	0.007 (−83.26%)	0.007 (−83.26%)	0.007 (−81.91%)
		550	0.007	0.008	0.008	0.007 (0.41%)	0.007 (0.41%)	0.007 (−0.59%)	0.007 (−0.43%)	0.007 (7.45%)
		1500	0.008	0.008	0.008	0.007 (−7.97%)	0.007 (−7.97%)	0.007 (−8.77%)	0.007 (−8.73%)	0.007 (−1.40%)
0.2	0.2	100	0.312	0.080	0.080	0.057 (−81.85%)	0.073 (−76.72%)	0.057 (−81.92%)	0.058 (−81.57%)	0.082 (−73.89%)
		550	0.143	0.080	0.080	0.057 (−60.39%)	0.075 (−47.31%)	0.056 (−60.93%)	0.058 (−59.76%)	0.082 (−32.75%)
		1500	0.092	0.080	0.080	0.057 (−38.18%)	0.071 (−22.19%)	0.056 (−39.04%)	0.058 (−37.18%)	0.082 (−18.73%)
	0.7	100	0.385	0.080	0.080	0.071 (−81.67%)	0.090 (−76.71%)	0.070 (−82.00%)	0.070 (−81.97%)	0.089 (−76.97%)
		550	0.178	0.080	0.080	0.071 (−60.20%)	0.081 (−54.40%)	0.070 (−60.87%)	0.070 (−60.81%)	0.089 (−49.93%)
		1500	0.097	0.080	0.080	0.070 (−27.51%)	0.081 (−16.63%)	0.070 (−28.61%)	0.070 (−28.49%)	0.089 (−8.65%)
2	0.2	100	1.715	0.677	0.687	0.567 (−66.94%)	0.565 (−67.08%)	0.558 (−67.48%)	0.558 (−67.48%)	0.601 (−64.93%)
		550	0.758	0.677	0.687	0.567 (−25.19%)	0.568 (−25.04%)	0.558 (−26.38%)	0.558 (−26.35%)	0.601 (−20.65%)
		1500	0.603	0.677	0.687	0.567 (−6.01%)	0.567 (−5.97%)	0.558 (−7.48%)	0.558 (−7.48%)	0.601 (−0.27%)
	0.7	100	4.153	0.696	0.696	0.704 (−83.06%)	0.707 (−82.98%)	0.695 (−83.27%)	0.694 (−83.29%)	0.745 (−82.06%)
		550	1.663	0.696	0.696	0.701 (−57.85%)	0.705 (−57.61%)	0.693 (−58.30%)	0.694 (−58.26%)	0.745 (−55.19%)
		1500	0.857	0.696	0.696	0.700 (−18.27%)	0.702 (−18.04%)	0.694 (−18.98%)	0.694 (−18.98%)	0.745 (−13.01%)

## Data Availability

The data that support the analysis of this study are openly available in https://doi.org/10.24432/C5F62R, accessed on 28 Februray 2025.

## Abbreviations

The following abbreviations are used in this manuscript:

i.i.d.	Independent and identically distributed
SAA	Sample average approximation
DRO	Distributionally robust optimization
KL	Kullback–Leibler
OLS	Ordinary least squares
DRCQP	Distributionally robust conditional quantile prediction
